# The intestinal microbiota of patients with type 2 diabetes is associated with the C-512T polymorphism of the FOXC2 gene

**DOI:** 10.3389/fendo.2025.1674135

**Published:** 2025-12-01

**Authors:** Lei Liang, Hongjiang Zhou, Xuxiang Zhang, Yandan Su, Ying Zhao, Xin Nian

**Affiliations:** 1 Department of Endocrinology, The First Affiliated Hospital of Kunming Medical University, Kunming, Yunnan, China; 2 Department of Endocrinology, Anhui Provincial Hospital, The First Affiliated Hospital, University of Science and Technology of China, Hefei, Anhui, China; 3 Department of Cultural Tourism and Wellness, Baoshan Vocational College, Baoshan, Yunnan, China; 4 Department of Laboratory Medicine, The First Affiliated Hospital of Kunming Medical University, Kunming, Yunnan, China

**Keywords:** intestinal microbiota, type 2 diabetes mellitus, *Collinsella*, FOXC2 gene, gene polymorphism

## Abstract

**Objective:**

To investigate the correlation between the C-512T polymorphism of the FOXC2 gene and intestinal microbiota in patients with T2DM.

**Methods:**

Peripheral blood and fecal specimens were collected from 170 patients with T2DM and tested for the C-512T polymorphism of the FOXC2 gene by DNA sequencing. Based on the genotype test results, 120 patients were finally included and divided into CC, CT, and TT groups (40 patients in each group). The structural composition and function of the intestinal microbiota were analyzed using 16S rRNA gene sequencing, and differences between groups of intestinal microbiota were assessed.

**Results:**

Alpha Diversity analysis suggested that there was no prominent difference in the abundance and diversity of intestinal microbiota among CC, CT, and TT groups. PCoA analysis in Beta diversity suggested that the composition of enteric flora might be different between CC and CT groups (*P* = 0.006078, *P* < 0.05); Anosim analysis showed that the differences in the structure of the intestinal microbiota were greater among the three groups than within the groups (*R* = 0.02203, *P* = 0.035, *P* < 0.05), with the greatest variability in the CC and TT groups. Finally, LefSe analysis revealed that Coriobacteriia, Clostridiales, Clostridiaceae, and Collinsella were significantly more abundant in the CC group than in the TT group.

**Conclusion:**

The C-512T polymorphism in the FOXC2 gene has been associated with the structural abundance and species composition of type 2 diabetic intestinal microbiota.

## 1 Introduction

Type 2 diabetes mellitus (T2DM) is a common chronic metabolic disease whose pathogenesis involves a complex interaction of genetic and environmental factors. Recent studies have found that the intestinal microbiota is not only involved in energy metabolism but also plays an important role in the development of T2DM and its complications by adjusting the host’s immune function, inflammatory response, and hormone secretion (1, 2). Dysregulation of intestinal microbiota can further exacerbate Insulin Resistance (IR) and hyperglycemia by promoting inflammatory responses, impaired barrier function, and metabolic disturbances (3). Genetic factors may play a key role in this process. A growing number of studies have confirmed that genetic factors may influence the composition and structure of human intestinal microbiota and that Single Nucleotide Polymorphisms (SNPs) of specific susceptibility genes are closely associated with the pathologic features of T2DM (4–6). Wang et al. analyzed 82 subjects from Germany and clarified that vitamin D receptors and other factors affect changes in the intestinal microbiota and that host genes influence the intestinal microbiota by about 10% (7). This also gives a theoretical basis for the study of genetics in the field of intestinal microbiota and T2DM.

Overexpression of the FOXC2 gene, a key metabolic regulatory gene, improves IR and reduces blood glucose levels (8). It has been shown that insulin sensitivity, lipid metabolism, and adipose tissue accumulation are associated with the C-512T polymorphism in the FOXC2 gene (9, 10). Our previous study found that the intestinal microbiota structure of FOXC2 C-512T CT genotypes in healthy populations differed in terms of α and β diversity, with significant variability in both CC and TT genotypes. The FOXC2 C-512T polymorphism may influence the composition of the human intestinal microbiota (11). Although studies have preliminarily revealed an association between intestinal microbiota and metabolic disorders, there is still a lack of direct authentication regarding whether genetic factors further impact on the development of T2DM by adjusting the intestinal microbiota.

This study focused on the field of intestinal microecology. Its primary aim was to analyze the differences in the structure and function of the intestinal microbiota among T2DM patients with different genotypes of the FOXC2 gene’s C-512T polymorphism. The ultimate goal of these efforts was to provide an academic basis for researching the synergistic effect between genetics and microbial communities.

## 2 Materials and methods

### 2.1 Research objectives

170 patients with T2DM hospitalized in the Department of Endocrinology of the First Affiliated Hospital of Kunming Medical University were selected according to the inclusion and exclusion criteria.

#### 2.1.1 Inclusion criteria

(1) The enrolled patients meet the WHO (1999) diagnostic criteria for diabetes mellitus: *a*. Diabetes mellitus symptoms (polydipsia, polyphagia, polyuria, weight loss, etc.) + random blood glucose ≥11.1 mmol/L or *b*. Fasting Plasma Glucose (FPG) ≥7.0 mmol/L or *c*. OGTT 2 hours Plasma Glucose (2hPG) ≥ 11.1 mmol/L. A diagnosis of diabetes is made when at least two blood glucose measurements exceed these values on different days; (2) Pancreatic β-cell autoantibodies were all negative; (3) Patients who were eligible for inclusion in the study of this experiment were informed of the specific purpose of the study, understood the significance of this study, voluntarily participated in this study and signed an informed consent form.

#### 2.1.2 Exclusion criteria

(1) Other special types of diabetes; (2) Pregnant and lactating women, patients with alcohol dependence or a history of substance abuse, and patients who have participated in other clinical studies in the 9 weeks before this trial; (3) Patients who have used glucose-lowering medications (GLP-1Rs, metformin, acarbose, etc.), antibiotics, probiotics, fiber supplements, and weight-loss medications within the last 2 months; (4) Patients taking nonsteroidal anti-inflammatory drugs, acid-suppressing drugs, and proton pump inhibitors for long periods; (5) Patients with gastrointestinal disorders (Ulcerative colitis, Crohn’s disease, and Short bowel syndrome, etc.); (6) Patients with physical or mental illnesses (Parkinson’s disease, Alzheimer’s disease, and Depression, etc.), or long-term use of antidepressants.

### 2.2 Specimen collection and preservation

Patients were fasted overnight for more than 8 hours, and 2 ml of peripheral venous blood was collected in the early morning in the fasting state, while fresh feces were collected from patients during early morning defecation. Label and quickly store in a -80 °C refrigerator within 10 minutes, avoiding repeated freezing and thawing during access.

### 2.3 FOXC2 C-512T polymorphic DNA sequencing

#### 2.3.1 DNA extraction and processing

Peripheral venous blood genomic DNA was derivative using a blood genomic DNA extraction kit (DP318 Centrifugal Column, TianGen Biotech Co., Ltd.) (11).

Pipette 1 mL of anticoagulated blood into a 2 mL centrifuge tube. Sequentially add 1 mL of cell lysis buffer CL, 200 μL of buffer GS, 200 μL of buffer GB, and 20 μL of Proteinase K. Centrifuge at Centrifuge S417R High-Speed Low-Temperature Centrifuge (12,000 rpm, Eppendorf AG, Germany) for 1 minute multiple times during this process. This step aims to lyse the cell walls and release DNA. Subsequently, add 350 μl of buffer BD. A flocculent precipitate may form at this stage. Transfer both the supernatant and the flocculent precipitate from the centrifuge tube into the CG2 adsorption column. Centrifuge at high speed (12,000 rpm) for 1 minute. Discard the supernatant from the collection tube and return the CG2 column to the tube. Add 500 μl of GDB buffer and 600 μl of PWB wash buffer to the CG2 column. Centrifuge twice at 12,000 rpm for 1 minute each. Discard the supernatant and return the CG2 column to the collection tube. Allow residual wash buffer to air-dry on the adsorbent material. Transfer the CG2 column to a 1.5 ml centrifuge tube. Add 70 μl of TB elution buffer to the center of the column, leaving space above the column. Incubate at room temperature for 2 minutes. Centrifuge at 12,000 rpm for 2 minutes. Collect the supernatant into the centrifuge tube. The DNA product extracted for the experiment is now in the centrifuge tube. Immediately store it at -80°C.

Finally, DNA concentration and purity were detected by Ultra-Micro Spectrophotometer Tnano-700 (Shanghai Tuohe Electromechanical Technology Co., Ltd.).

#### 2.3.2 PCR amplification and product characterization

According to the target genes, primer sequences for PCR reactions were designed using Primer Premier 5.0 software, forward (F) primer: 5′GCCGACGGATTCCTGCGCTC3′, reverse (R) primer: 5′CGCTCCTCGCTGGCTCCA3′ (Shanghai Sangong Bioengineering Co., Ltd.), and primers were formulated to a concentration of 10 umol/L of Liquid. The PCR reaction was performed in triplicate, incorporating positive and negative controls to ensure quality. Operate used the Model 9902 96-well PCR instrument (Allen-Bradley Company, USA).

PCR amplification reaction system: 1ul of forward primer and reverse primer (Shanghai Sangong Bioengineering Co., Ltd.), 14 ul of 2xTaq PCR Master Mix (Shanghai Toyobo Biotech Co., Ltd.), 2ul of DNA template, 32ul of sterile deionized water (China Biotopped Technology Co., Ltd.).

Reaction conditions: pre-denaturation (95°C) for 5 min for 1 cycle, denaturation (95°C) for 30s, annealing (63°C) for 30s, extension (72°C) for 30s. The 3 steps were performed for 35 cycles, followed by extension at 72°C for 10 min.

PCR product identification was performed using agarose gel electrophoresis (Biowest, Spain) (11).

#### 2.3.3 Sequencing analysis of PCR products

After obtaining the target PCR products, DNA sequence sequencing was performed and analyzed. The sequences of the DNA sequencing results were compared with the NCBI blast gene library, and the sequencing results were analyzed using Chromas software to complete the interpretation of the results.

### 2.4 Grouping of research subjects

**Figure 1:**
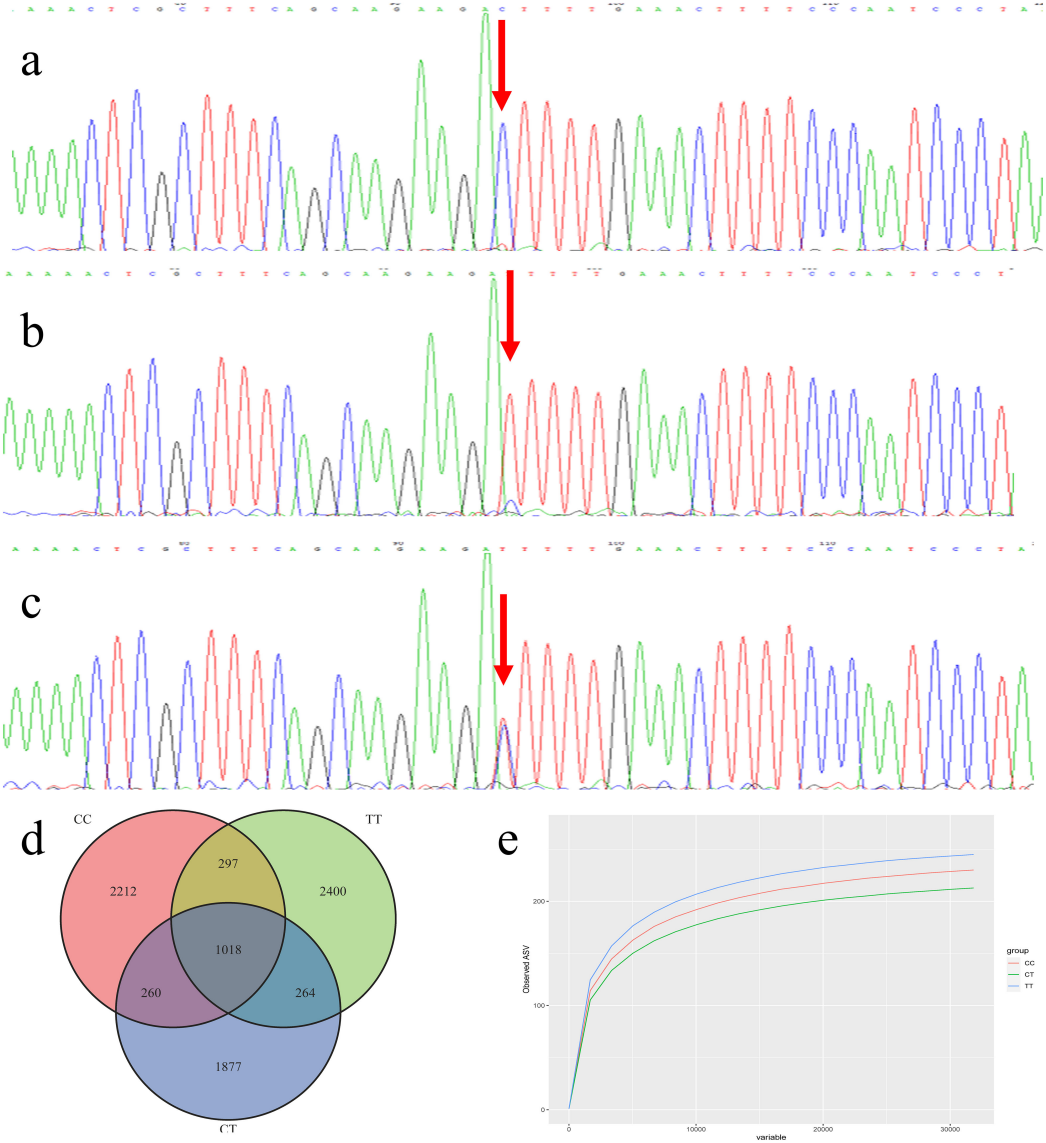
**(a)** Wild type (C/C); **(b)** Pure mutant (T/T); **(c)** Heterozygous mutant (C/T); **(d)** Venn diagram; **(e)** The Rarefaction curve.

According to the analysis of FOXC2 gene locus polymorphism, there are three genotypes: wild type (C/C) (**Figure 1a**), pure mutant (T/T) (**Figure 1b**), and heterozygous mutant (C/T) (**Figure 1c**). Therefore, we divided the experiment into three groups: the CC, TT, and CT groups.

### 2.5 Analysis of the diversity of intestinal microbiota

#### 2.5.1 Fecal genomic DNA extraction

Genomic DNA was extracted from fecal samples using the SDS method, and the purity and concentration of the DNA were examined using agarose gel electrophoresis at a concentration of 2% and Nanodrop (12).

#### 2.5.2 PCR product amplification

The extracted fecal genomic DNA samples were diluted to 1 ng/μl using sterilized water, and 10 ng of DNA samples were extracted as the template for this PCR reaction and placed in a centrifuge tube. The region selected for amplification this time was the V3-V4 region, and in order to ensure the efficiency and accuracy of amplification, specific primers were used (forward primer: 5’CCT-ACG-GGN-GGC-WGC-AG’3; reverse primer: 5’GGA-CTA-CHV -GGGG -TWT-CTA-AT’3) and efficient high-fidelity enzymes.

PCR amplification reaction system: High-Fidelity PCR Master Mix (TianGen Biochemical Technology Co., Ltd.) 15ul, primer 0.2uM, DNA template 10ng.

Reaction conditions: pre-denaturation (98°C) for 1min, denaturation (98°C) for 10s, annealing (50°C) for 30s, extension (72°C) for 30s, cycling for 30 times, and extension at 72°C for another 5min.

#### 2.5.3 Quantification and characterization of PCR products

1×loading buffer (containing SYBR green) was mixed evenly with the product of PCR in equal amounts and detected using agarose gel electrophoresis at a concentration of 2%. Observed under the ImageQuant LAS500 Gel Imaging System (General Electric Company), the samples with a main band brightness of 400bp-450bp were selected for the next step of the experiment. Aliquots were mixed according to the PCR product concentration, and the PCR products were prepared using the Tengen purification kit, and the purified products were used to prepare Illumina DNA libraries.

#### 2.5.4 Library construction and onboard sequencing

Construction of sequence detection libraries using the TIANSeq Rapid DNA Library Construction Kit (Illumina platform, TIANGEN Biotech). The established libraries were qualified by Qubit quantification and Agient2100 library assay, and then PE250bp sequencing was performed by the Illumina platform to obtain 250bp bipartite sequencing reads.

#### 2.5.5 Bioinformatics analysis

Bioinformatics analysis of high-throughput sequencing results was performed using R software. Information on species richness and evenness within the sample was obtained by analyzing ASVs/OTUs abundance calculations, Venn diagrams, and Alpha diversity; Multiple sequence comparisons of ASVs/OTUs were performed phylogenetic trees were constructed, and differences in community structure among groups were explored by PCoA and Anosim analyses in Beta diversity analysis; Specific differential strain analyses of the bacterial community structure of the three groups of samples were performed using LEfSe analysis (11).

### 2.6 Statistical analysis

Statistical analysis was implemented using SPSS 26.0 software. Measurement data were tested for normality, and bow to normal assignment was expressed as *M* ± *S* deviation, and comparisons between groups were made using analysis of variance (ANOVA); Disobedience to normal distribution was expressed as median (interquartile spacing *M* and 25%-75% percentile), and comparisons between groups were made using the kruskal-wallis *H* test. *P* < 0.05 indicates a statistically important difference and *P* < 0.01 indicates a statistically important difference.

## 3 Results

### 3.1 Sequencing results of the C-512T locus of the FOXC2 gene

170 cases met the criteria for experimental enrollment. The genes of each patient were analyzed by sequencing, including 46 patients in the CC group, 40 patients in the TT group, and 84 patients in the CT group. To verify whether the FOXC2 gene in our subjects conforms to the law of genetic equilibrium, we calculated a chi-square value of 0.0186 using the Hardy-Weinberg equilibrium formula. Consulting the chi-square distribution table, the critical value at 1 degree of freedom and P = 0.05 is approximately 3.841. Our chi-square value is significantly less than the critical value of 3.841. Therefore, the genotype distribution of the FOXC2 gene in our subjects complies with Hardy-Weinberg equilibrium (**Table 1**).

**Table 1:** Hardy-Weinberg equilibrium of the FOXC2 gene.

Genotype	Observed value(O)	Expected value(E)	(O-E)	(O-E)2	(O-E)2/E
CC	46	45.54	0.46	0.2116	0.0046
CT	84	84.88	-0.88	0.7744	0.0091
TT	70	39.56	0.44	0.1936	0.0049
Total	170	170			X2 = 0.0186

In order to reduce the bias between the three groups due to different sample sizes, we excluded some cases from the CC and CT groups using a random lottery method. Write the names of each subject in the CC and CT groups on separate slips of paper and shuffle them. Researchers randomly selected 40 subjects from each group to enroll in the study. Finally, sample size of each of the three groups included in the study was 40 cases, for a total of 120 cases.

### 3.2 Validation of grouping rationality

To validate our random sampling method, we used bioinformatics analysis to assess microbial community differences (before vs. after treatment) in the CC and CT groups, thereby eliminating potential bias associated with excluding partial samples.

Alpha diversity reflects the species diversity and abundance of the gut microbial community in a single sample. Commonly used metrics for alpha diversity include the Simpson indices and Shannon indices— which reflect community species diversity— and the Chao1 indices and Ace indices, which reflect species abundance (i.e., the number of species present). For samples with identical species abundance, higher Shannon and Simpson indices indicate greater species diversity.

**Figure 2:**
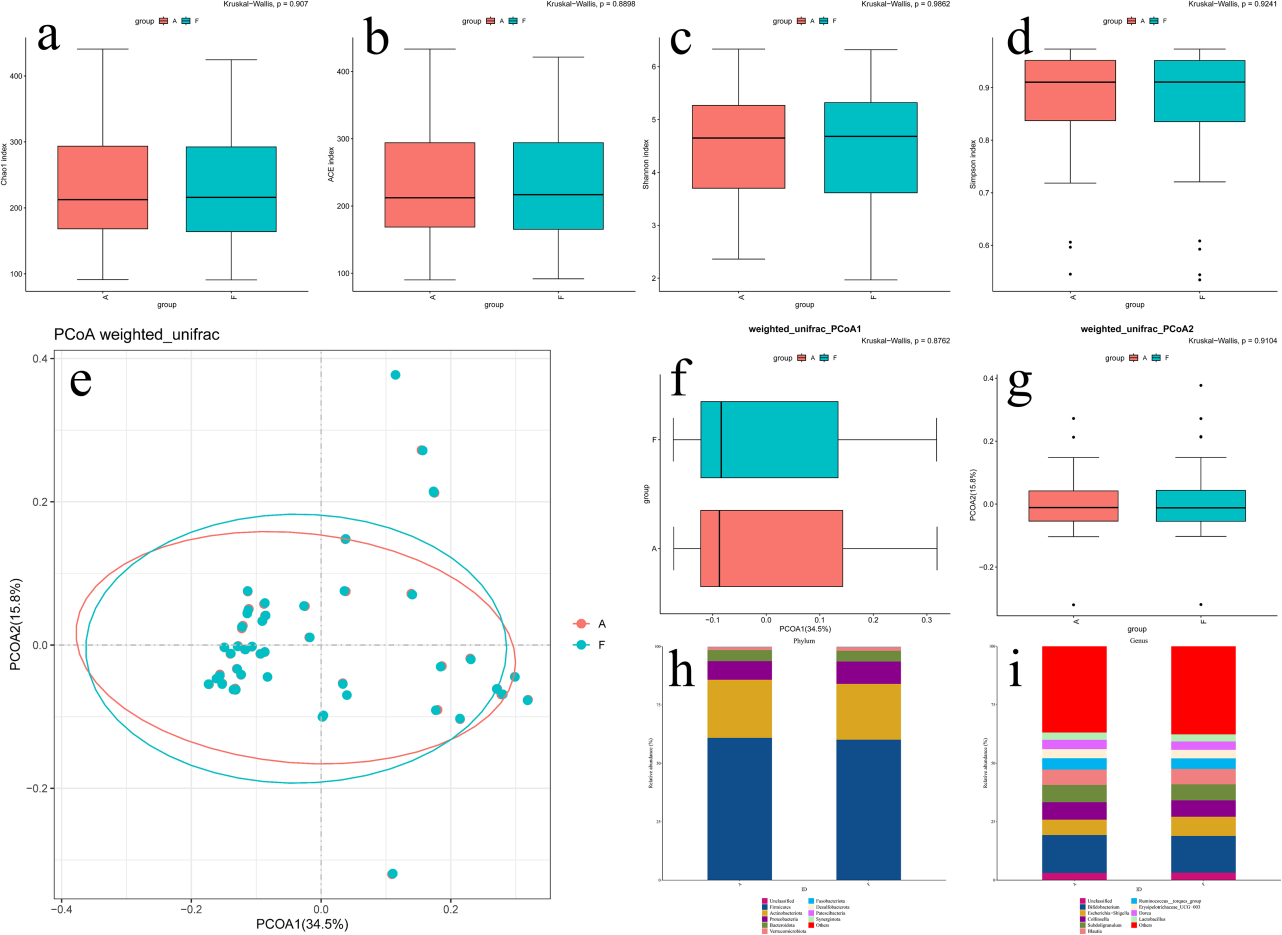
Bioinformatics analysis of gut microbiota before and after randomized grouping in CC group. **(a)** Chao1 index of Alpha Diversity; **(b)** Ace index of Alpha Diversity; **(c)** Shannon index of Alpha Diversity; **(d)** Simpson index of Alpha Diversity; **(e)** PCoA analysis of Beta Diversity; **(f)** PCoA1 quadrant box plots for PCoA analysis; **(g)** PCoA2 quadrant box plots for PCoA analysis; **(h)** Community structure diagrams at the phylum level; **(i)** Community structure diagrams at the genus level.

**Figure 3:**
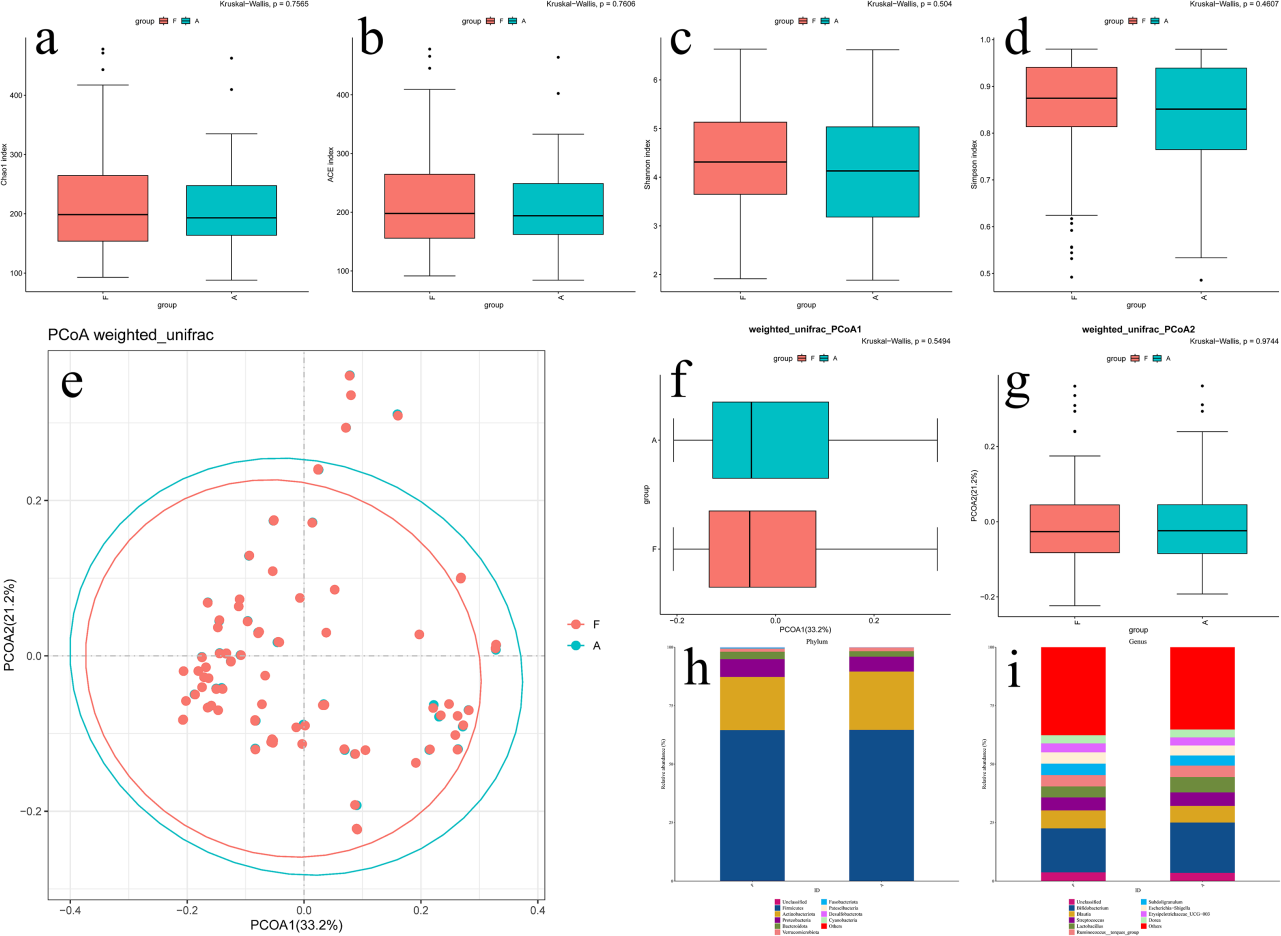
Bioinformatics analysis of gut microbiota before and after randomized grouping in CT group. **(a)** Chao1 index of Alpha Diversity; **(b)** Ace index of Alpha Diversity; **(c)** Shannon index of Alpha Diversity; **(d)** Simpson index of Alpha Diversity; **(e)** PCoA analysis of Beta Diversity; **(f)** PCoA1 quadrant box plots for PCoA analysis; **(g)** PCoA2 quadrant box plots for PCoA analysis; **(h)** Community structure diagrams at the phylum level; **(i)** Community structure diagrams at the genus level.

After randomly excluding some samples, alpha diversity analysis revealed no significant differences between the CC and CT groups in Chao1 indices (**Figures 2a**, **3a**), Ace indices (**Figures 2b**, **3b**), Shannon indices (**Figures 2c**, **3c**), and Simpson indices (**Figures 2d**, **3d**). This confirms that there were no significant differences in the abundance or diversity of gut microbiota between the CC group and the CT group before and after excluding certain samples.

Beta diversity, also referred to as between-habitat diversity, evaluates gut microbial community diversity across groups. By analyzing inter-sample diversity, it reveals differences in species abundance and diversity, thereby indicating whether significant microbial community variations exist.

Principal coordinates analysis (PCoA) extracts the most critical components and structures from multidimensional data via eigenvalue and eigenvector ordering (**Figures 2e**, **3e**). Differences in community species composition are represented by calculating inter-sample distances: samples with high similarity in community species composition tend to cluster together, while those with distinct compositions are widely separated. PCoA1 and PCoA2 analyses (conducted using the Weighted-Unifrac method) assess PCoA differences across two dimensional axes. After excluding the samples, we observed no significant changes in the composition or structure of the gut microbiota between the CC group (**Figures 2f, g**) and the CT group (**Figures 3f, g**).

Finally, we analyzed the dominant bacterial communities in the CC and CT groups—both before and after excluding partial samples—using species distribution bar charts at the phylum and genus levels. No significant changes were observed in the dominant microbial communities between the anterior and posterior intestinal segments.

#### 3.2.1 CC group

At the phylum level, dominant bacteria were Firmicutes, Actinobacteria, Proteobacteria, and Bacteroidetes (**Figure 2h**); at the genus level, they included *Bifidobacterium*, *Escherichia*, *Collinsella*, *Subdoligranulum*, *Blautia*, and *Ruminococcus* (**Figure 2i**).

#### 3.2.2 CT group

Dominant phyla were identical to those in the CC group (**Figure 3h**); at the genus level, dominant bacteria included *Bifidobacterium*, *Blautia*, *Streptococcus*, *Lactobacillus*, *Subdoligranulum*, and *Ruminococcus* (**Figure 3i**).

### 3.3 Comparison of clinical data and biochemical indicators of the study population

The clinical data and biochemical indexes of the three study groups were contrapositive, and there was no statistically important difference (*P*>0.05) in the comparison of body mass index (BMI), waist-hip ratio, Gender, age, High-density lipoprotein cholesterol (HDL), Low-density lipoprotein cholesterol (LDL), triglyceride (TG), and fasting blood glucose (FBG) in CC, TT, and CT groups (**Table 2**).

**Table 2:** Comparison of clinical data of the three groups.

Clinical Indicators	CC(n=40)	TT(n=40)	CT(n=40)	*F/χ^2^/H*	*P*
Age(years)	53.95 ± 12.70	53.73 ± 10.35	51.48 ± 9.56	0.625	0.537
Gender (Male)	23 (40)	23 (40)	17 (40)	2.406	0.3
BMI(kg/m^2^)	22.95 ± 2.80	23.66 ± 2.93	24.24 ± 3.27	1.851	0.162
WHR(cm)	0.8 ± 1.5	0.8 ± 1.43	0.8 ± 1.3	0.56	0.071
HDL(mmol/L)	1.13 ± 0.41	1.09 ± 0.35	1.05 ± 0.29	0.499	0.609
LDL(mmol/L)	2.60 ± 0.87	3.08 ± 1.20	2.72 ± 0.72	2.786	0.066
TG(mmol/L)	1.39(0.84,2.18)	1.65 (1.04,2.75)	1.7(1.06,2.54)	3.37	0.185
FPG(mmol/L)	5.98(4.96,8.99)	6.91(5.32,9.98)	6.18(4.78,8.23)	1.25	0.535

BMI, body mass index; WHR, waist-to-hip ratio; HDL, high-density lipoprotein; LDL, low-density lipoprotein; TG, triglyceride; FPG, fasting glucose, P < 0.05 difference was statistically significant.

### 3.4 16S rRNA sequencing analysis of intestinal microbiota

#### 3.4.1 Sequencing data processing results

Sequences obtained after sequence denoising or clustering through the QIIME2 dada2 analysis process are called Amplicon Sequence Variants (ASVs), which correspond to the Operational Taxonomic Units (OTUs) representative sequences. The abundance table of ASVs/OTUs sequences in a sample is called the feature table (13). After filtering, denoising, splicing, and decimalizing the sequence data, an average of 76,328 raw data were measured per sample. There are 76,319 data after filtering, 75,880 data after denoising, 74,321 sequences after splicing, and 71,591 valid data obtained after removing chimeras, with an effective rate of 93.79%.

The obtained valid data is then subjected to a de-duplication operation to obtain the de-duplication sequence ASVs. The above steps were analyzed separately for each library. The singletons ASVs (ASVs with a total sequence count of only 1 in the full sample) are then removed. Finally, to ensure comparability of species diversity between samples, standardization was performed using the diversity core-metrics-phylogenetic command in the QIIME2 software, with the depth of standardized data set to 95% of the minimum sample sequence size (11). The standardized samples were sequenced to a depth of 31846 and the number of ASVs was 8328.

The Venn diagram (**Figure 1d**) was used to show the number of ASVs that were common and individual between the stool samples of the three study groups, which visualized the overlap and independence of the number of ASVs between the samples. Of the 8328 different ASVs, 3787 ASVs were in the CC group, 3979 ASVs in the TT group, 3419 ASVs in the CT group, and 1018 ASVs were common to all 3 groups. This indicates that there were differences in the distribution of the intestinal microbiota among the three groups of samples, with the TT group having the highest number of ASVs and the most species-rich.

#### 3.4.2 Sample sequencing number saturation analysis

The Rarefaction Curve directly reflects whether the current sample ordering number is saturated or not, and indirectly reflects the species amplitude and diversity in the sample. As the Rarefaction curve smoothes out further up the curve, it indicates that the current amount of sequencing is sufficient and that more data volume will only operate a small number of new species. The Rarefaction Curve for each of the three sets of samples shows the number of ASVs observed (**Figure 1e**). Comparing the number of ASVs in different samples at the same sequencing depth provides some measure of how diverse each sample is.

The three groups of fecal samples were saturated with approximately 200–250 ASVs, with the highest number of ASVs in the TT group of samples, followed by the CC group, and the lowest in the CT group. The rarefaction curves of the three groups of samples in the figure gradually flatten out, indicating that the number of species sequenced and the sequencing depth are sufficient in this study. Even if the number of sequenced samples increases, the number of ASVs no longer increases significantly, and only a small number of new species (ASVs) will be generated, which will not affect the subsequent results. It indicates that even if some cases were excluded from the CC and CT groups during grouping in this study, there was no effect on the results of the abundance and diversity of the gut microbial community in each group.

#### 3.4.3 Species diversity analysis of intestinal microbiota communities

**Figure 4:**
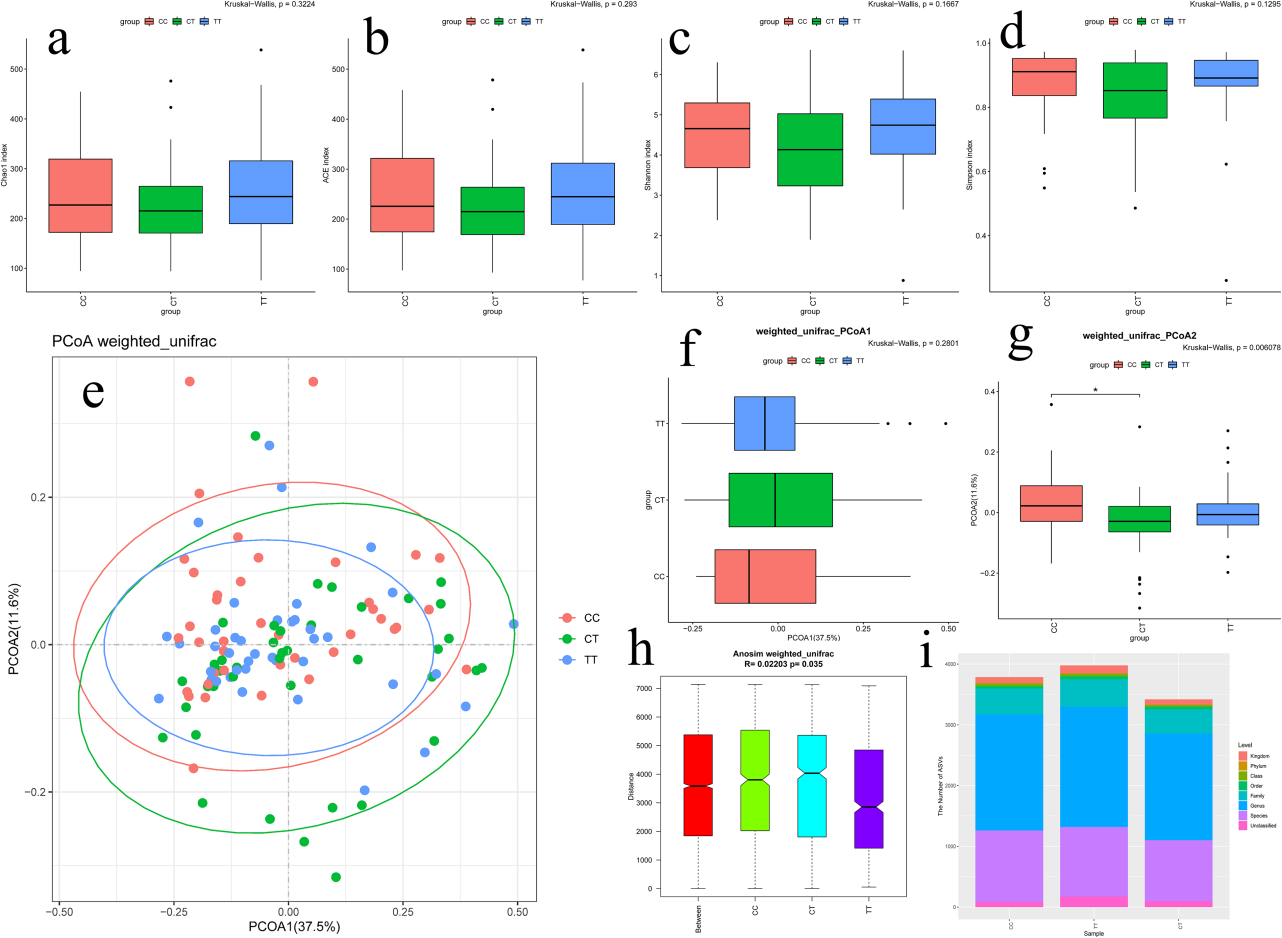
**(a)** Chao1 index of Alpha Diversity; **(b)** Ace index of Alpha Diversity; **(c)** Shannon index of Alpha Diversity; **(d)** Simpson index of Alpha Diversity; **(e)** PCoA analysis of Beta Diversity; **(f)** PCoA1 quadrant box plots for PCoA analysis; **(g)** PCoA2 quadrant box plots for PCoA analysis; **(h)** Anosim analysis box plots for Beta Diversity; **(i)** Tables of species abundance at different taxonomic levels for the three groups of samples.

There was no statistically significant difference (*P*>0.05) in the comparison of the gut microbiology samples of the three groups of patients on the four indices of Alpha diversity (**Figures 4a–d**). It was shown that there were no differences in microbial community species abundance and species diversity within the three groups of samples.

Weighted-Unifrac calculations showed a prominent difference (*P* = 0.006078, *P* < 0.05) in the spatial dimension of PCoA2 between the two groups, CC and CT (**Figures 4f, g**), suggesting that some of the gut microbial community species compositions differed between the two groups.

Anosim analysis was utilized to test whether the between-group differences were observably greater than the within-group differences to determine whether Beta Diversity was meaningful. According to the weighted unifrac algorithm analysis, it was tested whether the difference between the three groups was observably greater than the difference within the group. The results suggested that in the three groups of CC, TT, and CT, the difference between the groups was greater than the difference within the groups, and the CC group was the furthest away from the TT group (*R* = 0.02203, *P* = 0.035, *P* < 0.05), and the difference was statistically prominent (**Figure 4h**). This indicates that the species diversity of the gut microbial community differed among the three groups, again demonstrating the significance of the grouping in this study.

#### 3.4.4 Species annotation and its correlation analysis

By comparing the ASVs/OTUs sequences with the microbial reference Silva database, the species corresponding to each ASV/OTU could be clarified, and then the community composition of the three groups of samples was counted at the level of species, genus, family, order, class, and phylum, and the abundance table of species at each taxonomic level was generated (**Figure 4i**), and then used R language tools to map the community structure of the samples at each taxonomic level (14).

Species annotation was performed by comparison with the database Silva138, and statistical findings were made for the different taxonomic strata. Out of 8328 ASVs, the number of ASVs that could be annotated to the database was 95.33%. The proportion at the level of phylum is 91.43%, at the level of class is 91.12%, at the level of order is 89.88%, at the level of family is 88.43%, at the level of genus is 76.1%, and at the level of species is 28.25%.

At the kingdom level, the TT group was the richest; at the phylum level, the CC group was richer than the other two groups; at the class level, the CT group was less rich than the CC and TT groups; at the order level, the TT group was the richest, followed by the CT group, and lastly the CC group; at the family level, the TT group was the richest, followed by the CC group, and lastly the CT group; at the genus level, the TT group was the richest, followed by the CC group, and lastly the CT group; at the species level, the CC and TT groups are richer than the CT group (**Table 3**). In summary, the TT group of communities had the highest species richness, consistent with our Venn diagram results.

**Table 3:** Statistical table of species in each class of the three groups.

Sample	Kingdom	Phylum	Class	Order	Family	Genus	Species	Unclassified
CC	97	11	41	38	430	1910	1178	82
TT	133	5	41	52	452	1979	1143	174
CT	87	7	26	46	395	1758	1003	97

**Figure 5:**
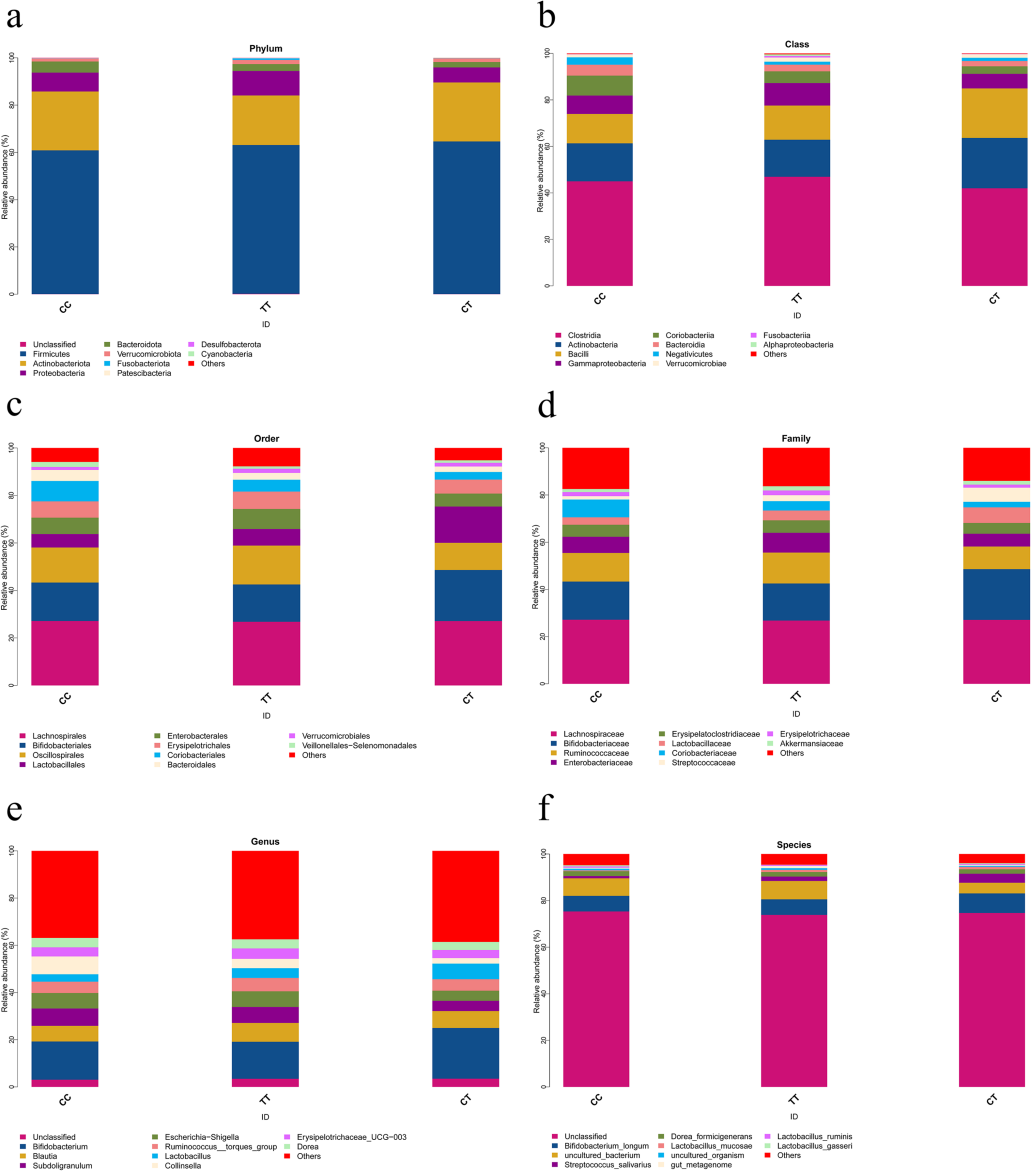
**(a)** Community structure diagrams of the three groups at the phylum level; **(b)** Community structure diagrams of the three groups at the class level; **(c)** Community structure diagrams of the three groups at the order level; **(d)** Community structure diagrams of the three groups at the family level; **(e)** Community structure diagrams of the three groups at the genus level; **(f)** Community structure diagrams of the three groups at the species level;.

The dominant flora that dominated the three groups of samples at the phylum level were Firmicutes, Actinobacteriota, and Proteobacteria (**Figure 5a**); The dominant flora that dominated the three groups of samples at the class level were Clostridia, Actinobacteria, and Bacilli (**Figure 5b**); The dominant flora that dominated the three groups of samples at the order level were Lachnospirales, Bifidobacteriales, and Oscillospirales (**Figure 5c**); The dominant flora that dominated the three groups of samples at the family level were Lachnospiraceae, Bifidobacteriaceae, and Ruminococcaceae (**Figure 5d**); The dominant flora that dominated the three groups of samples at the genus level were *Bifidobacterium*, *Blautia*, and *Subdoligranulum* (**Figure 5e**); The dominant flora that dominated the three groups of samples at the species level were *Bifidobacterium_longum*, *uncultured_bacterium*, and *Streptococcus_salivarius* (**Figure 5f**).

**Figure 6:**
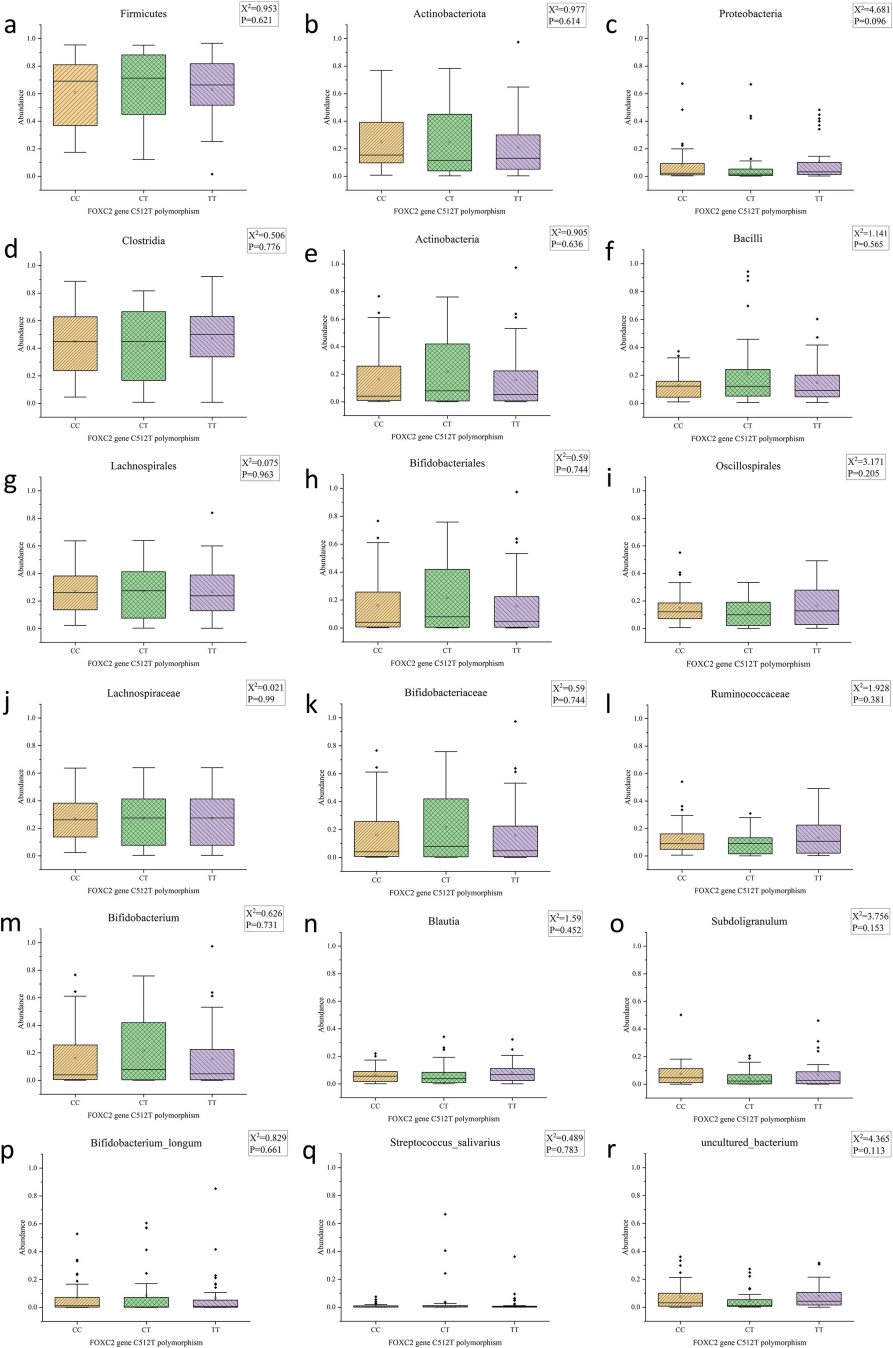
Boxplots showing differences in the top three dominant gut microbiota among different FOXC2 gene C512T variants across six taxonomic levels: phylum, class, order, family, genus, and species. **(a)** Abundance values of Firmicutes at the phylum level; **(b)** Abundance values of Actinobacteriota at the phylum level; **(c)** Abundance values of Proteobacteria at the phylum level; **(d)** Abundance values of Clostridia at the class level; **(e)** Abundance values of Actinobacteria at the class level; **(f)** Abundance values of Bacilli at the class level; **(g)** Abundance values of Lachnospirales at the order level; **(h)** Abundance values of Bifidobacteriales at the order level; **(i)** Abundance values of Oscillospirales at the order level; **(j)** Abundance values of Lachnospiraceae at the family level; **(k)** Abundance values of Bifidobacteriaceae at the family level; **(l)** Abundance values of Ruminococcaceae at the family level; **(m)** Abundance values of *Bifidobacterium* at the genus level; **(n)** Abundance values of *Blautia* at the genus level; **(o)** Abundance values of *Subdoligranulum* at the genus level; **(p)** Abundance values of *Bifidobacterium_longum* at the species level; **(q)** Abundance values of *Streptococcus_salivarius* at the species level; **(r)** Abundance values of *uncultured_bacterium* at the species level;

We further examined the abundance values of the top three bacterial species at each species level. The Kruskal-Wallis H rank sum test was employed for the analysis of differences. We found that across six taxonomic levels (phylum, class, order, family, genus, species), the top three dominant gut microbiota showed no significant differences in diversity at the C512T variant of the FOXC2 gene (**Figure 6**).

#### 3.4.5 LefSe analysis based on species abundance

LefSe is an analytical method based on Linear Discriminant Analysis (LDA) Effect size. The essence is to combine non-parametric statistical tests (Kruskal-Wallis multiple group test and Wilcoxon rank sum test) with LDA to screen for key biomarkers (e.g., key functional taxa/species), allowing us to identify traits of varying abundance as well as associated classes. It is not limited to analyzing differences between sample subgroups but can go deeper into different subgroups and pick key functional taxa/species that perform consistently across subgroups.

**Figure 7:**
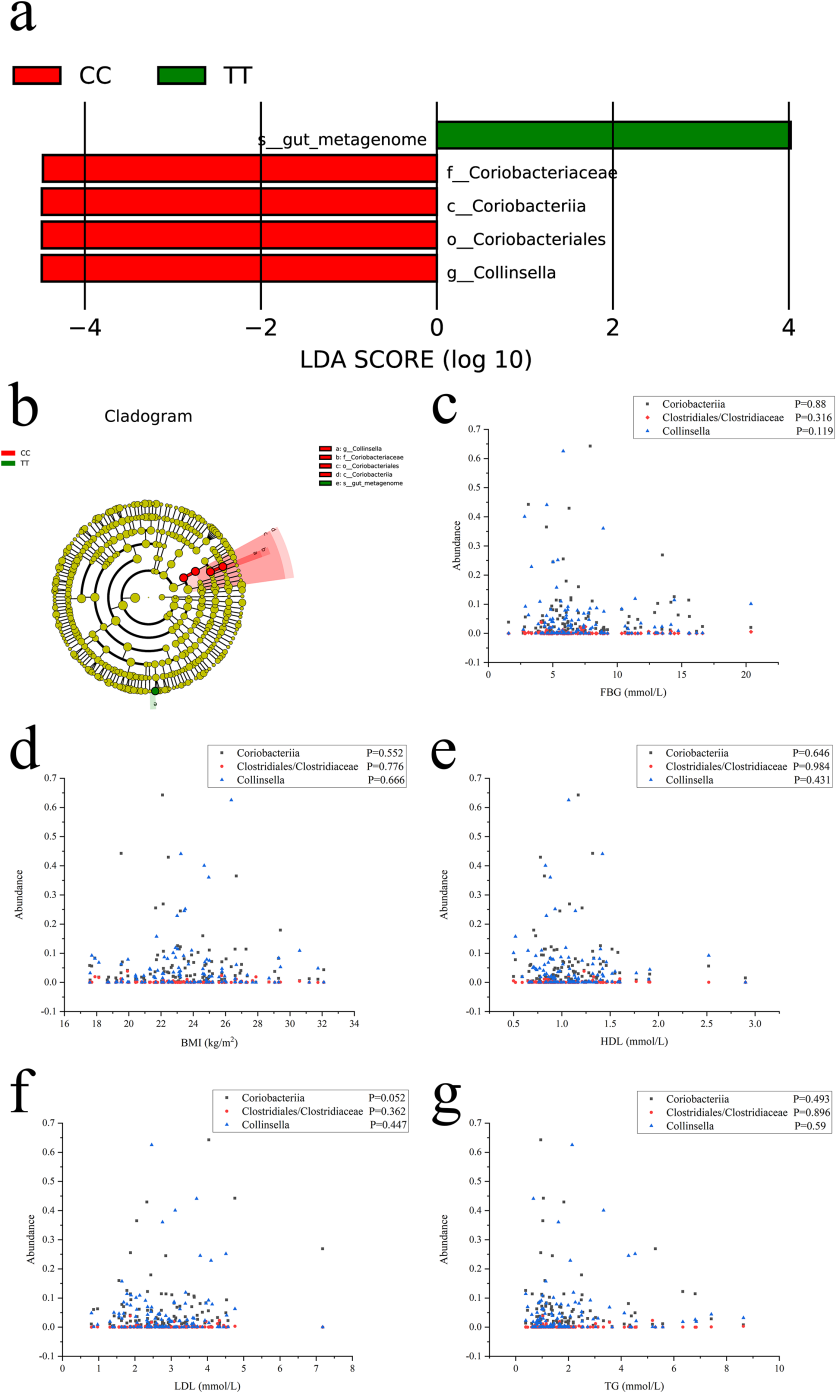
**(a)** Branching diagrams of evolutionary branching from the LefSe analyses; **(b)** Histograms of the distribution of LDA values; **(c)** Spearman correlation analysis between FBG and the abundance of Coriobacteriia, Clostridiales, Clostridiaceae, and *Collinsella*; **(d)** Spearman correlation analysis between BMI and the abundance of Coriobacteriia, Clostridiales, Clostridiaceae, and *Collinsella*; **(e)** Spearman correlation analysis between HDL and the abundance of Coriobacteriia, Clostridiales, Clostridiaceae, and *Collinsella*; **(f)** Spearman correlation analysis between LDL and the abundance of Coriobacteriia, Clostridiales, Clostridiaceae, and *Collinsella*; **(g)** Spearman correlation analysis between TG and the abundance of Coriobacteriia, Clostridiales, Clostridiaceae, and *Collinsella*;.

Based on the results of the detailed analysis of LefSe (**Figures 7a, b**). At the class level, Coriobacteriia had prominent species differences between the two groups of samples, the CC and TT groups, with higher numbers of colony ASVs/OTUs in the CC group than in the TT group; At the order level, Clostridiales had prominent species differences between the two groups of samples, the CC and TT groups, with higher numbers of colony ASVs/OTUs in the CC group than in the TT group; At the family level, Clostridiaceae showed prominent species differences between the two groups of samples, the CC and TT groups, with higher numbers of colony ASVs/OTUs in the CC group than in the TT group; At the genus level, *Collinsella* showed prominent species differences between the two groups of samples, the CC and TT groups, with higher numbers of colony ASVs/OTUs in the CC group than in the TT group.

### 3.5 Spearman correlation analysis

We further calculated the abundance values of Coriobacteriia, Clostridiales, Clostridiaceae, and *Collinsella* in each subject’s gut. We found that the abundance of Clostridiales at the order level was identical to that of Clostridiaceae at the family level. We used the Kruskal-Wallis H rank-sum test to further compare the abundance values of Coriobacteriia, Clostridiales, Clostridiaceae, and *Collinsella* in the intestines of the three groups of subjects. We found significant differences in Coriobacteriia (*X^2^* = 7.267, *P* = 0.026), Clostridiales (*X^2^* = 10.117, *P* = 0.006), Clostridiaceae (*X^2^* = 10.117, *P* = 0.006), and *Collinsella* (*X^2^* = 9.684, *P* = 0.008). The LefSe analysis results were validated.

Subsequently, we utilized Spearman correlation analysis to examine whether the abundance of Coriobacteriia, Clostridiales, Clostridiaceae, and *Collinsella* in the gut microbiota of patients with type 2 diabetes mellitus (T2DM) correlates with clinical indicators, including fasting blood glucose (FBG), body mass index (BMI), high-density lipoprotein (HDL), low-density lipoprotein (LDL), and triglycerides (TG).

Ultimately, our findings revealed the following (**Figure 7**):

The abundance of Coriobacteriia in the gut microbiota of T2DM patients showed no significant correlation with FBG (*r* = -0.022, *P* = 0.818), BMI (*r* = -0.056, *P* = 0.552), HDL (*r* = -0.043, *P* = 0.646), LDL (*r* = 0.18, *P* = 0.052), and TG (*r* = -0.065, *P* = 0.493).

The abundance of Clostridiales and Clostridiaceae in the gut microbiota of T2DM patients exhibited no significant correlation with FBG (*r* = -0.094, *P* = 0.316), BMI (*r* = -0.114, *P* = 0.776), HDL (*r* = -0.002, *P* = 0.984), LDL (*r* = 0.085, *P* = 0.362), and TG (*r* = 0.012, *P* = 0.896).

The abundance of *Collinsella* in the gut microbiota of T2DM patients demonstrated no significant correlation with FBG (*r* = -0.146, *P* = 0.119), BMI (*r* = 0.041, *P* = 0.666), HDL (*r* = -0.073, *P* = 0.431), LDL (*r* = 0.071, *P* = 0.447), and TG (*r* = 0.051, *P* = 0.59).

## 4 Discussion

Recent studies confirm intestinal microbiota plays an important role in energy metabolism. It also has a key role in T2DM development and progression. An unbalanced diet changes gut microbiota structure, letting pathogenic microorganisms become dominant. Early studies suggested T2DM development mainly links to high-fat diet-related overnutrition—such as excessive sugar, fat, and salt intake (15, 16). This disrupts enteric flora. A high-fat diet severely harms intestinal microbiota diversity and stability. It reduces beneficial flora and increases conditional pathogenic flora. This then triggers chronic low-grade gut inflammation and promotes IR development (17, 18). Later studies also show T2DM development closely relates to genetic factors. Interaction between gene susceptibility variants and dietary environment directly contributes to T2DM (19). However, the exact mechanism remains unclear. Revealing how susceptibility genes affect intestinal microbiota changes in T2DM is highly significant for guiding T2DM prevention and treatment.

The FOXC2 gene is located on chromosome 16 at the q24.1 locus. It belongs to the Forkhead box family and is also known as MFH-1.It plays a crucial role in human development and metabolism (11). The most common FOXC2 gene variant identified to date (C-512T) is located in the 5′ untranslated region (20). Our previous study found a link between the C-512T polymorphism of the FOXC2 gene and glucose-lipid metabolism abnormalities in diabetes. IR was significantly lower in TT-type patients than in CC and CT-type patients (*P* = 0.03) (21). Ridderstråle et al. also made a finding. They found the T allele correlated with enhanced IR (*P* = 0.007) in women (22). Another study noted something too. The C allele, along with CC and CT phenotypes, was markedly overexpressed in men with T2DM (23). All of these studies suggest that the CC-type population is likely to have a higher incidence of T2DM than the TT-type population.

Our study found that while there was no marked difference in Alpha Diversity among patients with CC, CT, and TT genotypes in C-512T of the FOXC2 gene, there was a difference in Beta Diversity between CC and CT genotypes (*P* = 0.006078, *P* < 0.05). It suggests that the C-512T polymorphism of the FOXC2 gene does not affect the abundance and diversity of the intestinal microbiota, but there may be differences in the composition of gut microorganisms between CC and CT types. Interestingly, we further analyzed the Beta Diversity rows using Anosim analysis. While the results showed that the composition of the enteric flora did differ among the three groups, the CC and TT groups were the furthest apart and more differentiated (*R* = 0.02203, *P* = 0.035, *P* < 0.05).

To further investigate species-specific differences in the gut microbiota associated with the FOXC2 gene C-512T polymorphism in patients with type 2 diabetes, we employed LefSe analysis for the final assessment. Results revealed a striking distinction in intestinal microbiota between the CC and TT genotype groups: the CC group exhibited significantly higher abundances of Coriobacteriia, Clostridiales, Clostridiaceae, and *Collinsella* compared to the TT group. LefSe-based analysis of species evolutionary cladograms indicated that these four microbial taxa occupy distinct hierarchical positions in the evolutionary tree. We propose that these taxa may collectively contribute to the dominance of *Collinsella* observed in the CC group. The FOXC2 gene C-512T may influence T2DM through the intestinal microbiota.

Interestingly, we used Spearman correlation analysis to examine the relationship between clinical indicators in T2DM patients and the abundance of Coriobacteriia, Clostridiales, Clostridiaceae, and *Collinsella*. We found no significant correlation between FBG, BMI, HDL, LDL, and TG levels and the abundance values of these bacterial groups. We believe this may be due to the lack of data from healthy individuals and the small sample size in our study.

There is growing evidence that the FOXC2 gene plays an important role in metabolism, especially lipid metabolism. The FOXC2 gene is closely associated with the development of IR and T2DM (24). Overexpression of the FOXC2 gene in transgenic mice exhibited increased insulin sensitivity, decreased insulin, triglyceride levels, and, blood glucose. And relative resistance to diet-induced obesity and IR (25). FOXC2 has an important role in insulin regulation, and FOXC2 mRNA levels in visceral fat and muscle are negatively interrelated with the degree of IR (8). In the state of obesity, massive expansion of adipose tissue, hypertrophy of white adipocytes, and macrophage infiltration in adipose tissue activates the complex of Toll-like Receptor 4 (TLR4) with LPS, which induces the production of pro-inflammatory adipocytokines, ultimately leading to IR (9).

Notably, Gan et al. showed the FOXC2 gene inhibits LPS-mediated adipocyte inflammation. It also promotes white adipose tissue browning by positively regulating leptin signaling and the STAT3-PRDM16 complex (9). Further studies note FOXC2 acts in preadipocytes. After high-fat diet-induced FOXC2 expression, Akt/mTORC1 and ERK/mTORC1 pathways in adipose precursor cells are activated. This enhances precursor cell proliferation, inhibits their differentiation into mature adipocytes, and suppresses precursor cell apoptosis. The result is elevated cyclin E expression and reduced p27 and p53 levels (26).

Additionally, FOXC2 synergizes with α-melanin-stimulating hormone (α-MSH). This promotes fatty acid oxidation in both brown and white adipose tissue (10). Lidell et al. identified FOXC2’s role in regulating metabolism and mitochondrial function. FOXC2 overexpression in adipocytes increases mitochondrial generation and fusion, raises mitochondrial number, and leads to an elongated mitochondrial morphology. These changes boost energy expenditure and fatty acid oxidation (27).

Early growth response protein 1 (Egr-1)—a negative regulator of FOXC2—is highly expressed in obesity and IR models. Knocking down Egr-1 upregulates FOXC2 expression, producing effects similar to FOXC2 overexpression. This improves hyperlipidemia and IR symptoms (5). Zhang et al. found insulin induces FOXC2 protein expression. It does so by regulating the activity of the FOXC2 gene’s C-512T promoter. This inhibits the differentiation of adipose tissue-derived MSCs, reduces adipocyte quantity, and improves IR (28). The FOXC2 gene’s C-512T T/T genotype correlates with increased visceral adiposity-related mRNA expression. It also links to higher insulin sensitivity and lower triglyceride levels (8). In conclusion, the FOXC2 gene influences T2DM development via multiple complex mechanisms.

Notably, recent studies have found that FOXC2 also affects diabetes by regulating intestinal epithelial function. Deletion of the FOXC2 gene leads to impaired intestinal barrier, and dysregulation of intestinal microbiota and triggers metabolic disorders and systemic inflammation, which in turn exacerbates the onset and progression of T2DM (6). This interaction between intestinal microbiota and genetic factors demonstrates a broader role for FOXC2 in metabolic regulation. However, due to the paucity of relevant studies. There is currently insufficient theoretical basis to support this idea, but it is still a promising area of research.

Our study aims to investigate differences in the gut microbiota of type 2 diabetes patients with distinct FOXC2 gene C512T polymorphisms. We seek to identify pathogenic bacteria that may contribute to the onset of type 2 diabetes, thereby providing a theoretical foundation for subsequent mechanistic research.

The normal intestinal microbiota consists mainly of anaerobic bacteria, and common groups include Verrucomicrobia, Firmicutes, Actinobacteria, Bacteroidetes, Fusobacteria, and Proteobacteria. Of these, the first four phyla accounted for 98% of the total intestinal microbiota, with proportions of Actinobacteria 3%, Proteobacteria 8%, Bacteroidetes 23%, and Firmicutes 64% (1). Specifically at the genus level, *Vulgatus*, *Clostridium*, and *Bifidobacterium* are the dominant fecal flora, while *Desulfovibrio*, *Enterobacterium*, *Lactobacillus*, and other bacteria are the secondary dominant flora of the adult gut (29). Despite the great variation between bacterial species, more than 99% of the total bacterial population in the human gut consists mainly of 30 to 40 bacterial species, with smaller proportions of other bacteria (30). Predominated by Firmicutes and Bacteroidetes, based on their functions, intestinal bacteria can be categorized as beneficial, harmful, and conditionally pathogenic (30, 31), which are closely related to the health status of the human body (32).

The intestinal microbiota has significant anthropogenic characteristics that depend on a variety of factors such as medications, diet, genetics, lifestyle, and hygiene (33–35). Larsen et al. demonstrated for the first time that there is a prominent difference between the intestinal microbiota of diabetic patients and the normal population, with diabetic patients showing a marked decrease in the quantities of *Clostridium* and Firmicutes and a marked increase in the quantities of *Bacteroides* and *Prevotella (*2). Wu et al. found that the amount of *Bacillus* in the feces of diabetic patients was much lower than that of healthy individuals (3). LEKA et al. found that the quantities of intestinal *Bifidobacterium* was significantly lower in T2DM patients than in healthy individuals, while the number of *Enterococcus* was significantly higher (36). Zhang et al. showed that the reduction of Verrucomicrobia may be a potential marker for the development of T2DM, which was significantly reduced in both early and advanced T2DM patients (37).

*Collinsella* is one of the two representative genera of Actinobacteria, the other being *Bifidobacterium*. Unlike *Bifidobacterium*, which is the generally known probiotic, *Collinsella* has been associated with a variety of diseases in humans (38). *Collinsella* can induce rheumatic diseases by promoting the expression of IL-17 cells, thereby affecting the immune function and impairing the integrity of the intestinal barrier (39, 40). In addition, *Collinsella* abundance was significantly increased in T2DM and *Collinsella* abundance was also positively correlated with Gestational diabetes mellitus (GDM) (41, 42). Interestingly, our study found that in the guts of T2DM patients, the abundance of *Collinsella* was not significantly correlated with fasting blood glucose levels. But our study confirms that the abundance of the *Collinsella* is significantly elevated in individuals with the CC genotype of the FOXC2 gene. However, these individuals exhibit a higher incidence of T2DM (23). This finding is consistent with previous research results, and also suggests a potential close association among these three factors.

Unfortunately, this study did not include insulin measurements at the time of subject recruitment, precluding the calculation of subjects’ HOMA-IR. Analyzing fasting blood glucose alone cannot distinguish whether elevated blood glucose arises from insufficient insulin secretion or insulin resistance. This may result in a one-sided assessment of the mechanisms underlying abnormal glucose metabolism. Future research is therefore needed to clarify the correlation between *Collinsella* and clinical indicators in patients with type 2 diabetes mellitus.

While the exact mechanism by which intestinal microbiota affects T2DM remains unclear, its metabolites are currently recognized as key mediators. Qin et al. used intestinal metagenomic sequencing to analyze diabetics’ feces. They found moderate intestinal ecological disturbances in diabetics: butyrate-producing *Rothia* and *Prevotella* decreased, while conditional pathogens like *Closnostachys rosea* and *Bacteroides stercoris* increased (19). Similarly, Eckburg et al. found higher levels of butyrate-producing bacteria (e.g., *Prevotella*, *Eubacterium*, *Clostridium*) in healthy subjects. In contrast, conditional pathogens like *Desulfovibrio* and *Escherichia coli* were significantly elevated in diabetics (43).

These changes correlate with more active sugar and branched-chain amino acid transport across intestinal cell membranes in diabetics, reduced butyrate synthesis, and increased oxidative stress. T2DM patients’ enteric flora is marked by decreased butyrate-producing bacteria and dysbiosis. This promotes an inflammatory environment, raises expression of microbial genes linked to oxidative stress, lowers expression of vitamin synthesis-related genes, and increases lipopolysaccharide (LPS) concentration. These effects further disrupt the intestinal barrier and boost intestinal permeability (44).

Similarly, *Collinsella* also impairs the intestinal barrier function. It reduces intestinal mucosal mucus synthesis, epithelial cell regeneration, and the tight junction protein zonulin (ZO-1) (45). Peng et al. made a key finding. They found *Collinsella* may raise type 2 diabetes mellitus (T2DM) incidence by upregulating certain human metabolic pathways. These include biosynthesis of type II polyketide products, Epstein-Barr virus infection, histidine metabolism, and sulfur metabolism. It also downregulates two other metabolic pathways: butanoate metabolism and phenylalanine metabolism (46). Additionally, *Collinsella* also induce IR and promote fat accumulation by stimulating the production of LPS (47). Positive correlation with pro-inflammatory responses and adverse outcomes (48). Overall, Collinsella also has the potential to become a signature intestinal microbiota for T2DM.

## 5 Conclusion

In conclusion, we analyzed the effect of the C-512T polymorphism of the FOXC2 gene on the structure of intestinal microbiota in patients with T2DM. We found that the three genotypes of the FOXC2 gene were differentially associated with the intestinal microbiota, with the abundance of Coriobacteriia, Clostridiales, Clostridiaceae, and *Collinsella* in the gut microbes of patients with CC-type T2DM being significantly higher than that of patients with TT-type T2DM. These results provide new insights into the FOXC2 gene in type 2 diabetes and the compositional structure of the intestinal microbiota. *Collinsella*, as a potential key pathogen in type 2 diabetes, may be involved in the metabolic processes of the FOXC2 gene in patients with type 2 diabetes. We will continue to explore the specific mechanisms by which Collinsella influences the pathogenesis of type 2 diabetes through the FOXC2 gene.

## 6 Limitations

Our study also has some limitations. The first is the issue of sample size, which leads to significant difficulties in subject inclusion in clinical studies due to the plethora of factors that can influence intestinal microbiota. Secondly, since this study was a preliminary exploratory study, it did not further explore the underlying molecular mechanisms. However, our study provides theoretical support for subsequent genetic studies of T2DM with intestinal microbiota-related therapies.

## Data Availability

The datasets presented in this study can be found in online repositories. The names of the repository/repositories and accession number(s) can be found below: https://www.ncbi.nlm.nih.gov/, PRJNA950549.
